# Case of Vomica Cured by the Operation for Empyema

**Published:** 1814-08

**Authors:** 


*For the Medical and Physical Journal.*

### *Case of Vomica cured by the Operation for Empyema.*

A MAN, 43 years of age, of a sanguineous temperament, after having experienced on the 24th of October pains in his kidneys with general indisposition, was seized on the 28th with a stitch in the side, which in a few hours propagated itself to all the right side of the chest, accompanied by a dry cough and fever. These symptoms were not relieved by the treatment employed, but continued eleven days, at which time they were exchanged for copious perspiration, and the expectoration of very thick and black matter. During the remainder of the month these last symptoms diminished. The perspirations in particular were confined to the pit of the stomach, but at the same time the patient began began to experience cold chills and slight hectic fever. On the 1st of December, being on horseback, after violent coughing, he spit blood in large quantity. From this time he brought up a large quantity of pus and grumous blood, sometimes by expectoration, and at others by a kind of vomiting. At the end of this month two little inflammatory tumours, of the size of a pigeon's egg, appeared on the margin of the arms, which were speedily resolved, and replaced by others of the same kind on the external and lower part of the fore-arm. These disappeared like the former, but there remained some time a swelling of the wrist and palm of the hand of the right side.

During the month of January the patient continued to get worse. On the 23d he was seen by M. Jaymes, when the following particulars were noted. He was greatly extenuated; the nails were livid; the eyes and cheeks were hollow, the latter of a reddish brown colour; his appetite was gone; tongue white; and he could lay only on the left side. There was no diarrhœa. He had slow fever; heat in the throat; cough; purulent expectoration; difficulty of breathing. A portion of the integuments on the right side was emphysematous, and a manifest gurgling was heard in the corresponding cavity. Between the fifth and sixth true ribs a small tumour appeared, which projects at every fit of coughing, and disappears in their interval. The lower extremities were œdematous. M. Jaymes, convinced by the assemblage of these symptoms of the existence of fluid in the right cavity of the chest, advised the patient to submit to the operation for empyema.

On the 1st of February, three months after the attack of the pleurisy, he proceeded as follows:—The patient being placed on the left side, M. Jaymes made, with an ordinary bistory, an incision in the projecting part of the integuments, viz. between the fifth and sixth rib. Some air escaped. By a second stroke of the knife an incision was made perpendicularly down to the superior margin of the sixth rib, and, accompanied by the fore finger, the surgeon divided the intercostal muscles and the pleura, for the space of half an inch. At the opening of the chest, the air escaped in abundance, but at first nothing else, although a sound introduced into the cavity left no doubt of its having been opened. Nevertheless, three hours after, the patient having made great efforts at expectoration in consequence of the cough, about a pint of fluid of a reddish colour, mixed with membranous flocculi, suddenly escaped from the wound, and forced away the dressings with which it was covered. On the following morning the matter was white and puriform. The three following days four more pints were evacuated by the wound. About two pints afterwards came away during the same space. The pus now became serous, and diminished in quantity. On the twentieth day the discharge was about a spoonful at each dressing, and completely disappeared at the fifty-fifth day from the operation.

During this time the patient was dressed twice in twenty-four hours. Injections of honey mixed with water were used for six weeks, which frequently, instead of returning by the wound, were evacuated by expectoration. From the moment of the operation the patient began to show signs of amendment. From this time there was an evident diminution in the expectoration emphysema, heat in the throat, and slow fever. The appetite returned, and the patient could lay on his right side, which was before impossible. On the fourth and the seventh he had favourable perspirations, and again on the twentieth, twenty-second, and about the thirty-seventh day of the operation.—*Journale de Medicine.*

